# 

**Published:** 1886-03

**Authors:** 


628 W. Lake St., CHICAGO, ILL., March 3d, 1886.

Editor of AMERICAN JOURNAL OF DENTAL SCIENCE:

*Dear Sir.*—The following resolution was adopted at the January meeting of the Chicago Dental Society and the Corresponding Secretary instructed to transmit a copy to your Journal for publication.

RESOLVED, That this Society endorse the action of the conference at Buffalo, as regards the International Medical Congress.

(The action is embodied in the followiug resolution:)

RESOLVED, That we as members of the dental profession deem it inexpedient to recommend the organization of a Section of Dental and Oral Surgery in the International Medical Congress of 1887 under the present circumstances.

P. J. KESTER, *Cor. Sec.* Chicago Dental Society.

### ILLINOIS STATE DENTAL SOCIETY.

The twenty-second annual meeting will be held at Rock Island, Ill., beginning Tuesday, May 11th, and continuing four days. Dentists in this and adjoining States are cordially invited to attend.

J. W. WASSAL, *Secretary*,
208 Dearborn Ave., Chicago, Ill.

---

### Editorial, Etc.

### To Readers and Correspondents.

Communications intended for publication, and exchange journals and papers should be addressed to the Editor of the American Journal of Dental Science, 259 N. Eutaw St., Baltimore, Md. All letters relating to the business of the Journal, or containing cash remittances, to be directed to Messrs. Snowden & Cowman. Articles for publication should be received by the editor at least three weeks previous to the first month on which the number in which it is designed for them to appear, is issued.

☞ The American Journal of Dental Science will contain fifty pages of reading matter in each number, making a volume of about

*Six Hundred* pages,

EXCLUSIVE OF ADVERTISEMENTS.

